# Identification of Selective Inhibitors of the *Plasmodium falciparum* Hexose Transporter PfHT by Screening Focused Libraries of Anti-Malarial Compounds

**DOI:** 10.1371/journal.pone.0123598

**Published:** 2015-04-20

**Authors:** Diana Ortiz, W. Armand Guiguemde, Alex Johnson, Carolyn Elya, Johanna Anderson, Julie Clark, Michele Connelly, Lei Yang, Jaeki Min, Yuko Sato, R. Kiplin Guy, Scott M. Landfear

**Affiliations:** 1 Department of Molecular Microbiology & Immunology, Oregon Health & Science University, Portland, OR, United States of America; 2 Department of Chemical Biology & Therapeutics, St. Jude Children’s Research Hospital, Memphis, TN, United States of America; University of Melbourne, AUSTRALIA

## Abstract

Development of resistance against current antimalarial drugs necessitates the search for novel drugs that interact with different targets and have distinct mechanisms of action. Malaria parasites depend upon high levels of glucose uptake followed by inefficient metabolic utilization via the glycolytic pathway, and the *Plasmodium falciparum* hexose transporter PfHT, which mediates uptake of glucose, has thus been recognized as a promising drug target. This transporter is highly divergent from mammalian hexose transporters, and it appears to be a permease that is essential for parasite viability in intra-erythrocytic, mosquito, and liver stages of the parasite life cycle. An assay was developed that is appropriate for high throughput screening against PfHT based upon heterologous expression of PfHT in *Leishmania mexicana* parasites that are null mutants for their endogenous hexose transporters. Screening of two focused libraries of antimalarial compounds identified two such compounds that are high potency selective inhibitors of PfHT compared to human GLUT1. Additionally, 7 other compounds were identified that are lower potency and lower specificity PfHT inhibitors but might nonetheless serve as starting points for identification of analogs with more selective properties. These results further support the potential of PfHT as a novel drug target.

## Introduction

Malaria represents a major global health challenge and is estimated to be responsible for ~216 million infections per year resulting in ~655,000 deaths in 2010 (World Malaria Report 2011, http://www.who.int/malaria/world_malaria_report_2011/en/). Drug resistance continues to present a major obstacle to control of this disease, leading to the use of combination therapies [1]. The front line therapy is currently Artemisinin Combination Therapy, but this treatment is now threatened by the emergence of slow responding strains of the parasites [2,3]. Hence there is an urgent need to develop novel therapies that target different pathways from those disrupted by current drugs [4]. Furthermore, there is great interest in drugs that could be effective against multiple stages of the malaria life cycle [5] to prevent development of disease, to control disease pathology, and to prevent transmission from one infected individual to the next.

One remarkable aspect of the physiology of malaria parasites is their complete dependence upon glucose uptake and glycolytic metabolism [6]. Because the parasites do not express a mitochondrial pyruvate dehydrogenase [7], they rely completely on glycolysis for glucose catabolism, thus generating only two ATP molecules per glucose. The Krebs Cycle and oxidative phosphorylation are not engaged for production of ATP from glucose. This inefficient use of glucose forces the parasite to transport large amounts of glucose to sustain viability and thus makes the parasite especially dependent on glucose uptake. Hence, inhibiting glucose import from the host’s blood may be a novel therapeutic strategy.

In 1999 Krishna and colleagues [8] cloned and functionally expressed the gene for the hexose/glucose transporter PfHT from *Plasmodium falciparum*. This permease is a member of the Facilitative Glucose Transporter or SLC2 family [9]. However, PfHT is highly divergent in sequence from all human orthologs, with GLUT1, the most closely related human ortholog, sharing only 28% amino acid identity with PfHT. This modest relatedness suggested that it might be possible to identify compounds that inhibit PfHT with high affinity without strongly inhibiting the orthologous human GLUTs. Subsequently, the Krishna laboratory demonstrated that the D-glucose analog 3-*O*-((undec-10-en)-1-yl)-D-glucose, also referred to as compound 3361, was able to selectively inhibit uptake of glucose by PfHT versus human GLUT1 [10]. Specifically, the K_i_ for glucose uptake by PfHT was 53 μM while no significant inhibition of GLUT1 was observed at a 1 mM concentration of compound 3361. Compound 3361 also inhibited growth of intraerythrocytic *P*. *falciparum* parasites *in vitro* with an IC_50_ value of 15.7 μM, and it induced a 40% reduction in parasitemia of mice infected with *P*. *berghei* parasites when administered at a dose of 25 mg/kg (*i*.*p*.) twice daily [10]. These results strongly suggested that PfHT provides an essential route for nutrient uptake in blood stream stage parasites.

Subsequently, genetic evidence was advanced by two groups [11,12] demonstrating the inability to delete the *PfHT* gene, unless parasites had been first transfected with an episomal copy of the gene to provide complementation. These results supported the notion that PfHT is an essential glucose transporter for intraerythrocytic parasites. Additionally, studies applying compound 3361 to hepatic stage and ookinetes of *P*. *berghei* demonstrated strong inhibition of viability of both these liver and mosquito stages of the malaria life cycle [11,13], implying that PfHT and its orthologs in other species of malaria are indeed essential in multiple stages of parasite development.

As indicated by Krishna and colleagues [14–16], these observations suggest that inhibiting the parasite PfHT without impairing function of human SLC2 transporters such as GLUT1 might be a promising strategy for development of drugs. Transporters represent the targets for ~13% of currently FDA-approved oral drugs with known targets in humans [16], establishing that permeases are often ‘druggable’ proteins likely to contain binding pockets for small molecules that are usually unrelated in structure to their natural permeants. Although compound 3361 represents one such selective inhibitor, it is not a drug-like compound and is not considered a lead for drug development [16]. Hence, it is important to identify other non-sugar compounds that selectively inhibit PfHT and might be advanced toward novel therapeutic agents. One approach to identifying novel PfHT inhibitors is to screen libraries of drug-like compounds for those that selectively inhibit PfHT with high affinity. The challenge in implementing this approach is to develop an assay for transporter function that can be carried out in a high-throughput screening strategy. We have previously demonstrated the both PfHT and GLUT1 can be heterologously expressed in a glucose transporter null mutant (Δ*lmxgt1-3*) of the parasitic protozoan *Leishmania mexicana*, and that these transgenic parasites (Δ*lmxgt1-3*[p*PfHT*] or Δ*lmxgt1-3*[p*GLUT1*]) are auxotrophic for glucose [17]. In the current study we have employed a cell proliferation assay, using the DNA-binding dye SYBR Green, to screen two ‘focused’ libraries of compounds with demonstrated ability to inhibit growth of *P*. *falciparum* parasites *in vitro*. The first library is the Tres Cantos antimalarial compound set (TCAMS) that consists of 13,533 compounds (https://www.ebi.ac.uk/chemblntd) that inhibit growth of *P*. *falciparum* 3D7 intraerythrocytic parasites by ≥80% at 2 μM concentration [18]. The second library is the Malaria Box collection of 400 compounds [19] with demonstrated antimalarial activity (http://www.mmv.org/research-development/malaria-box-supporting-information) that was obtained from the Medicines for Malaria Venture. Following the primary screen for compounds that inhibited growth of Δ*lmxgt1-3*[p*PfHT*] transgenic parasites, we applied secondary screens that directly measured glucose uptake to identify the limited subset of primary hits that inhibited growth of this reporter cell line by selectively inhibiting glucose import through PfHT compared to GLUT1. This multistep approach identified two compounds that were high affinity inhibitors of PfHT and low affinity inhibitors of GLUT1. Analysis of analogs uncovered a third compound with similar properties. Additionally, 7 low potency, low selectivity inhibitors of PfHT also emerged. These hits provide compounds with potential for drug development against the essential *P*. *falciparum* hexose transporter PfHT.

## Materials and Methods

### High Throughput Screening

#### i. Parasite proliferation assay employing SYBR Green

To monitor proliferation of reporter cell lines in the presence of library compounds, 15 μL of DME-L medium [20] containing 5 mM glucose and 10% heat inactivated fetal bovine serum was dispensed into each well of 384-well microplates (black polystyrene, clear bottom, tissue culture treated, Corning) with a Matrix Wellmate liquid dispenser (Thermo Scientific). Stock compounds dissolved in dimethyl sulfoxide (DMSO) were pin-transferred (V&P Scientific) into the microplate to the desired final concentration using an automated robot arm. 15 μL of the reporter cell line, e.g., Δ*lmxgt1-3*[p*PfHT*], at 2 x 10^6^/mL was added per well with the Wellmate dispenser. Microplates were incubated (Liconic) at 28°C and 5% CO_2_ for 72 h. After incubation, 10 μL of the reading solution (5X SYBR Green prepared from a commercial 100X stock, SIGMA, 5% Triton-X in PBS) was added per well. Each plate was shaken at 1000 rpm for one min, incubated further at room temperature for 20 min, and then fluorescence was read (excitation 485 nm, emission 535 nm) with the Envision plate reader (PerkinElmer). All data processing and visualization, as well as chemical similarity and substructure analysis, was performed using custom programs written in the Pipeline Pilot platform (Accelrys, v.7.0.1) and the R program [21].

#### ii. Glucose uptake assay in 96-well plate format

To measure uptake of glucose in a medium throughput format, 100 μL/well of PBS was added to 96-well filter plates (Millipore) with the Wellmate dispenser. Control wells included either 0 mM (positive control) or 20 mM (negative control) of the competitive inhibitor fructose in PBS. Compounds were added with the Biomek FX automated system. 90 μl of a cell suspension (1.1 x 10^8^ cells/mL) was added to each well with the Wellmate dispenser and left at room temperature for 5 minutes before adding 10 μL of the substrate (4 mM [^3^H] D-glucose at 50 μCi/mL) to provide a final glucose concentration of 200 μM. After a 5 min incubation, the uptake reaction was stopped by adding 50 μL/well of 4% formaldehyde, followed by incubation for ~5 min. Cells were filtered and washed with a vacuum manifold (Millipore). Plates were dried overnight, 100 μL of scintillation fluid were added, and plates were then sealed and read on a TopCount NXT HTS from PerkinElmer. Data quality and analysis was performed using the GUItars program [22] and sigmoidal curve fitting with Pipeline Pilot platform (Accelrys, v. 7.0.1).

### Kinetic Analysis of Nutrient Uptake in the Presence of Inhibitors

#### i. Compounds and radiolabel

Compounds 7, 8, and 16–21 were purchased from Ambinter (Orléans, France). Compound 1 and 10–15 were purchases from ChemDiv (San Diego, CA). All purchased compounds were subjected to quality control testing by their respective manufacturers. Dimethyl sulfoxide (DMSO) for solubilizing compounds was obtained from Sigma-Aldrich.

Radiolabelled [2-^3^H(N)] D-glucose (21.2 C_i_/mmol), [5-6-^3^H] uridine (38 C_i_/mmol) (2 μC/mL), [2,8-^3^H] hypoxanthine (31.8 C_i_/mmol) (10 μC/mL) and [2,3,4,5-^3^H] L-proline (60 C_i_/mmol) (4 μC/mL) were purchased from Moravek Biochemicals

#### ii. Uptake assays

Glucose uptake assays employed logarithmic phase *L*. *mexicana* promastigotes of the Δ*lmxgt1-3* hexose transporter null mutant strain expressing either PfHT or GLUT1 from an episomal expression vector [17]. Parasites (1x10^7^) were resuspended in 100 μL phosphate buffered saline, pH 7.4. Prior to uptake, parasites were pre-incubated for 5 min with inhibitor, and uptake was initiated by adding 100 μL of 200 μM [^3^H] D-glucose diluted to 4.0 μC_i_/mL. Uptake was terminated, typically after 1 min, by centrifuging the parasites through a layer of dibutyl phthalate, as described [23]. Control uptake assays employing [^3^H] hypoxanthine, [^3^H] uridine, and [^3^H] L-proline were performed similarly. Analysis of kinetic data was performed using Graph Pad Prism 4.0b software (Graph Pad).

### Growth Inhibition of *P*. *falciparum* and Human Foreskin Fibroblasts *in vitro*

#### i. Biological assay

Two *P*. *falciparum* strains were used in this study and were provided by the MR4 Unit of the American Type Culture Collection (ATCC, Manassas, VA). Those two strains were the chloroquine sensitive strain 3D7 and the K1 strain that is resistant to chloroquine, pyrimethamine, and sulfadoxine.

#### ii. Proliferation of parasites and EC_50_ determinations

Asynchronous parasites were maintained in culture based on the method of Trager [24]. Parasites were grown in presence of fresh group O-positive erythrocytes (Key Biologics, LLC) in Petri dishes at a hematocrit of 4–6% in RPMI based media (RPMI 1640 supplemented with 0.5% AlbuMAX II, 25 mM HEPES, 25 mM NaHCO_3_ (pH 7.3), 100 μg/mL hypoxanthine, and 5 μg/mL gentamycin). Cultures were incubated at 37°C in a gas mixture of 90% N_2_, 5% O_2_, 5% CO_2_. For EC_50_ determinations, 20 μL of RPMI 1640 with 5 μg/mL gentamycin were dispensed per well in an assay plate (Corning 384-well microtiter plate, clear bottom, tissue culture treated, catalog no. 8807BC). 40 nL of compound, previously serially diluted in a separate 384-well white polypropylene plate (Corning, catalog no. 8748BC), was dispensed to the assay plate by hydrodynamic pin transfer (FP1S50H, V&P Scientific Pin Head) and then 20 μL of a synchronized culture suspension (1% rings, 4% hematocrit) was added to each well, thus making a final hematocrit and parasitemia of 2% and 1%, respectively. Assay plates were incubated for 72 h, and the parasitemia was determined by a method previously described [25]: Briefly, 10 μL of the assay solution in PBS (10X SYBR Green I, 0.5% v/v triton X-100, 0.5 mg/mL saponin) was added to each well. Assay plates were shaken for 1 min, incubated in the dark for 90 min, and read with an Envision (Perkin Elmer) spectrophotometer at Ex/Em of 485 nm/535 nm. EC_50_s were calculated with a custom program (RISE, Robust Investigation of Screening Experiments) using a four-parameter logistic equation.

#### iii. Mammalian cell drug susceptibility assay

The BJ cell line (human foreskin fibroblasts), which express GLUT1 [26], was purchased from the ATTC and cultured according to recommendations. 1000 exponentially growing cells were plated per well (25 μL) in white polystyrene flat bottom sterile 384-well tissue culture treated plates (Corning), and incubated overnight at 37°C in a humidified 5% CO_2_ incubator. DMSO inhibitor stock solutions were pin-transferred (V&P Scientific) the following day. Plates were placed back in the incubator for 72 h incubation and equilibrated at room temperature for 20 min before addition of 25 μL Cell Titer Glo (Promega) to each well. Plates were shaken on an orbital shaker for 2 min at 500 rpm. Luminescence was read after 15 min on an Envision plate reader (Perkin Elmer). EC_50_ values were calculated with a custom program (RISE, Robust Investigation of Screening Experiments) using a four-parameter logistic equation.

### Physical Properties and *in vitro* Pharmacokinetics

#### i. Solubility assay

The solubility assay [27,28] was carried out on a Biomek FX lab automation workstation (Beckman Coulter, Inc.) using μSOL Evolution software (pION, Inc.) as follows: 10 μL of compound stock was added to 190 μL of 1-propanol to make a reference stock plate. Next, 5 μL of this reference stock plate was mixed with 70 μL of 1-propanol and 75 μL of PBS (pH 7.4) to make the reference plate, and the UV spectrum (250–500 nm) of the reference plate was read. Then, 6 μL of 10 mM test compound stock was added to 600 μL of PBS, pH 7.4, in a 96-well storage plate and mixed. The storage plate was sealed and incubated at room temperature for 18 h. The suspension was then filtered through a 96-well filter plate (pION Inc.). Next, 75 μL of filtrate was mixed with 75 μL of 1-propanol to make the sample plate, and the UV spectrum of the sample plate was read. Calculations were done using μSOL Evolution software based on the area under the curve (AUC) of the UV spectrum of the sample plate and the reference plate. All compounds were tested in triplicate.

#### ii. Parallel artificial membrane permeability assay (PAMPA)

A parallel artificial membrane permeability assay (PAMPA) [28,29] was conducted on a Biomek FX lab automation workstation (Beckman Coulter, Inc.) with PAMPA evolution 96 command software (pION Inc.) as follows: 3 μL of 10 mM test compound stock was mixed with 600 μL of PBS (pH 7.4) to make diluted test compound. Then 150 μL of diluted test compound was transferred to a UV plate (pION Inc.), and the UV spectrum was read as the reference plate. The membrane on a preloaded PAMPA sandwich (pION Inc.) was painted with 4 μL of GIT lipid (pION Inc.). The acceptor chamber was then filled with 200 μL of acceptor solution buffer (pION Inc.), and the donor chamber was filled with 180 μL of diluted test compound. The PAMPA sandwich was assembled, placed on the Gut-Box controlled environment chamber and stirred for 30 min. The aqueous boundary layer was set to 40 μm for stirring. The UV spectrum (250–500 nm) of the donor and the acceptor were read. The permeability coefficient was calculated using PAMPA Evolution 96 Command software (pION Inc.) based on the AUC of the reference plate, the donor plate, and the acceptor plate. All compounds were tested in triplicate.

#### iii. Liver microsomes stability assay

The NADPH regenerating agent solutions A (catalog#: 451220) and B (catalog#: 451200) and mouse liver microsomes (CD-1, mixture of male, catalog#:452701, and female, catalog#: 452702) were obtained from BD Gentest. The microsomal stability assay was carried out as described [29,30]. For each test compound, the mouse liver microsomal solution was prepared by adding 58 μL of concentrated mouse liver microsomes (20 mg/mL protein concentration) to 1.756 mL of 0.1 M potassium phosphate buffer (pH 7.4) containing 5 μL of 0.5 M EDTA to make a 0.6381 mg/mL (protein) microsomal solution. Each test compound (2.2 μL of 10 mM DMSO solution) was added directly to 1.79 mL of mouse liver microsomal solution and 90 μL was transferred to wells in 96-well plates (0, 0.25, 0.5, 1, 2, and 4 h time points each in triplicate). The NADPH regenerating agent was prepared by mixing 0.113 mL of NADPH regenerating agent solutions A, 0.023 mL of solution B and 0.315 mL of 0.1 M potassium phosphate buffer (pH 7.4) for each tested compound. To each well of the 96-well plate, 22.5 μL of the NADPH regenerating agent was added to initiate the reaction, and the plate was incubated at 37°C for each time point (0, 0.25, 0.5, 1, 2, and 4 h time points each in triplicate). The reaction was quenched by adding 225 μL of cold acetonitrile containing warfarin (4 μg/mL) as internal control to each well. All of the plates were centrifuged at 3220 g for 20 min and the supernatants (100 μL) were transferred to another 96-well plate for analysis on UPLC—MS (Waters Acquity UPLC linked to Waters Acquity Photodiode Array Detector and Waters Acquity Single Quadrupole Mass Detector) on an Acquity UPLC BEH C_18_ 1.7 μm (2.1x50 mm) column by running 90–5% gradient for water (0.1% formic acid) and acetonitrile (0.1% formic acid) in 2 minutes. The area under the single ion recording (SIR) channel for the test compound divided by the area under the SIR for internal control at 0 time concentration was considered as 100% to calculate remaining concentration at each time point. The terminal phase rate constant (ke) was estimated by linear regression of logarithmic transformed concentration versus time, where ke = slope x(-ln10). The half-life t_1/2_ was calculated as ln2/ke. The intrinsic clearance (CL_int,app_) = (0.693/in vitro t_1/2_) x (1mL incubation volume/0.5 mg of microsomal protein) x (45 mg microsomal protein/gram of liver) x (55 g of liver/kg body weight) [31,32].

## Results

### Development of an HTS assay against PfHT

To identify selective inhibitors of PfHT, it was necessary to develop an HTS compatible assay that could identify compounds that inhibit the glucose uptake activity of PfHT. For this purpose, we expressed both PfHT and GLUT1 in a glucose transporter null mutant of *L*. *mexicana*, Δ*lmxgt1-3*, and grew the transgenic parasites in high glucose (5 mM) DME-L medium [20] that lacks the alternate carbon source proline [33] so that proliferation of the reporter lines would be completely dependent upon uptake of glucose through either PfHT or GLUT1 [17]. Proliferation of reporter strains was monitored by DNA content using the fluorescent dye SYBR Green [25]. Initial plate uniformity assays (http://htsc.wustl.edu/Aids/NCGC_Assay_Guidance_Manual.pdf) of the PfHT-expressing line in triplicate 384-well plates, using high (no phleomycin), medium (1.4 μM phleomycin) and low (1 mM phleomycin) growth conditions, resulted in Z’ values [34] of 0.87, 0.87, and 0.89 (a Z’ value of 1.0 would represent a perfect assay without error) and Coefficients of Variation for the high growth conditions of 3.3, 3.3, and 2.9% for each of the triplicate plates, indicating a robust HTS assay. A subsequent scaling screen of the ~2000 compound MicroSource Discovery Spectrum Collection resulted in a Z value of 0.81 and a Coefficient of Variation of 5.0%. The average Z value for plates in the primary screens of the HTS described below was 0.8.

### Primary, secondary, and tertiary screens of the TCAMS library

**Fig 1:**
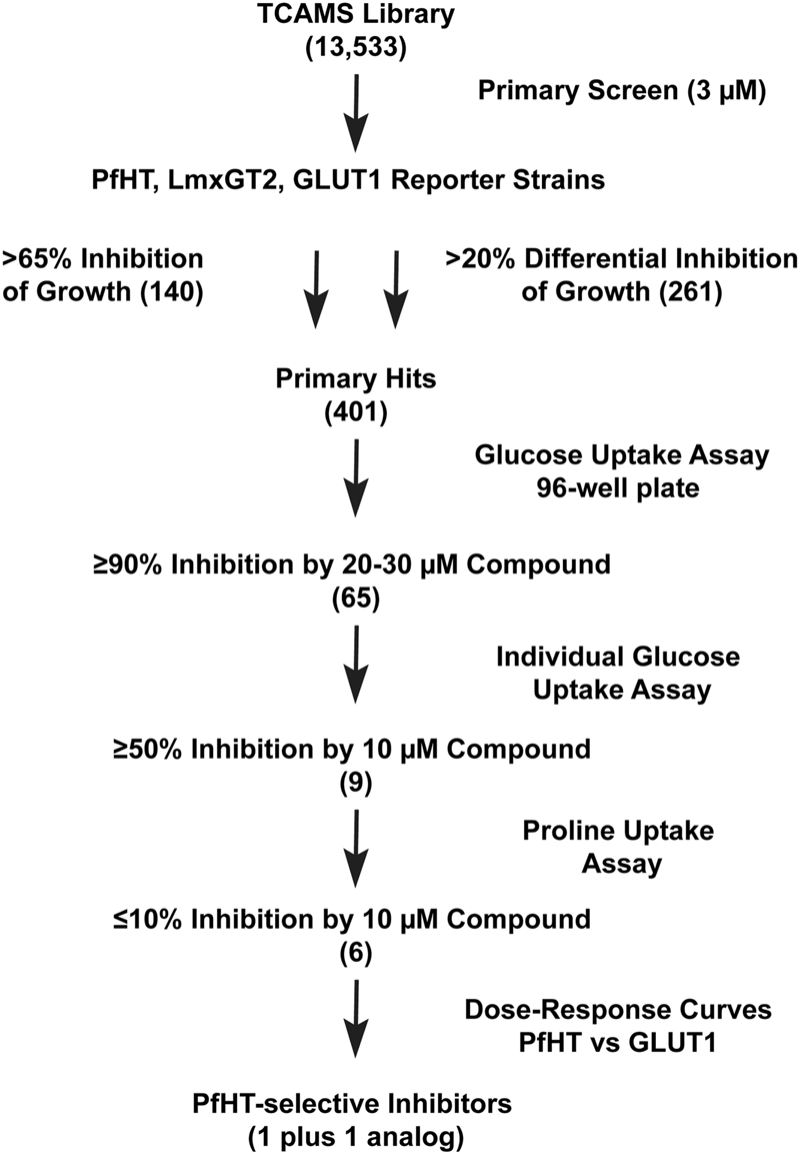
Flow chart for screen of TCAMS library. The TCAMS library of 13,533 compounds with demonstrated growth inhibitory activity against intraerythrocytic *Plasmodium falciparum* parasites was screened by sequential criteria. The steps included: 1) proliferation inhibitory screen (>65% inhibition at 3 μM concentration or >20% differential inhibition among the three strains) of PfHT, LmxGT2, and GLUT1 reporter strains to produce 401 primary hits; 2) 96-well plate assays for compounds (20–30 μM) that inhibited uptake of 200 μM [^3^H] D-glucose by ≥90%; 3) individual uptake assays for compounds (10 μM) that inhibited glucose uptake by ≥50%; 4) individual uptake assays for compounds (10 μM) that inhibited uptake of 100 μM [^3^H] L-proline by ≤10%; 5) dose-response curves for compounds that selectively inhibited uptake of glucose through PfHT versus GLUT1 (1 compound plus 1 additional hit that emerged from analysis of analogs). Numbers in parentheses represent the number of positive hits obtained after each sequential step.

We first performed a primary screen of the 13,533 compound TCAMS library [18]. A flow chart for the screen is outlined in Fig 1. Since this screen was part of a project to capture hexose transporter inhibitors for multiple parasites, compounds were screened in duplicate at 3 μM concentration employing 3 transgenic Δ*lmxgt1-3* lines expressing PfHT, the *L*. *mexicana* hexose transporter LmxGT2, and human GLUT1. Primary hits met one of two criteria for the average of duplicate samples: i) >65% inhibition of growth of each reporter line (140 hits), a cut off chosen on the basis of receiver-operator characteristics [35,36], ii) >20% differential inhibition of one line versus another (261 hits). This dual strategy will capture both broad-spectrum hexose transporter inhibitors and those selective for individual permeases.

**Fig 2:**
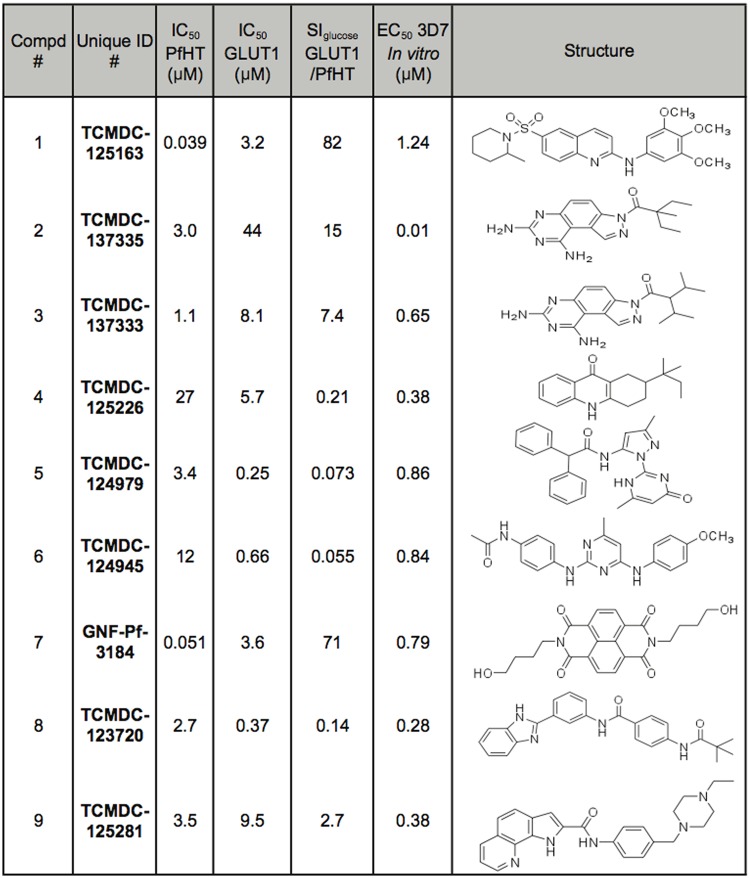
Compounds from the TCAMS (1–6) and Malaria Box (7–9) libraries that were selective inhibitors of glucose, but not proline, transport. Dose-response curves for inhibition of uptake of 100 μM [^3^H] D-glucose by each compound were performed for PfHT and GLUT1, and the IC_50_ values are tabulated. SI_glucose_ indicates Specificity Index for glucose uptake and is IC_50_ for GLUT1/IC_50_ for PfHT. The reported EC_50_ values (PubChem web site, http://pubchem.ncbi.nlm.nih.gov/) for inhibition of growth of *P*. *falciparum* strain 3D7 intraerythrocytic forms by each compound are also tabulated.

The 401 primary hits were subsequently screened in glucose uptake assays performed in 96-well plates. The 65 compounds that inhibited uptake of 200 μM [^3^H] D-glucose by ≥90% when applied at 20–30 μM concentrations were designated secondary hits. Because of the relatively large scatter in uptake assays performed in a plate format, these 65 secondary hits were subsequently tested at 10 μM concentration in more accurate and reproducible individual glucose uptake assays [17], performed in triplicate, and the 9 that inhibited uptake by ≥50% were designated tertiary hits. These hits were also tested for inhibition of uptake of 100 μM [^3^H] L-proline to remove compounds that non-specifically inhibit membrane transport processes (≥10% inhibition of proline uptake at 10 μM compound), giving 6 validated hits that are selective inhibitors of glucose transport (Compounds 1–6 in Fig 2).

### Screen of the Malaria Box Library

Screening was also performed on the 400 compound Malaria Box library. Since this library was much smaller than the TCAMS, it was screened initially at a concentration of 20–30 μM using the 96-well glucose uptake assay to provide 47 secondary hits with ≥90% inhibition of uptake by the PfHT reporter strain. These hits were rescreened in individual glucose and proline uptake assays at 10 μM to reveal 3 validated hits (Compounds 7–9 in Fig 2) that gave 75–100% inhibition of glucose uptake without significant inhibition of proline uptake.

### Compounds that Inhibit Glucose Uptake Through PfHT

**Fig 3:**
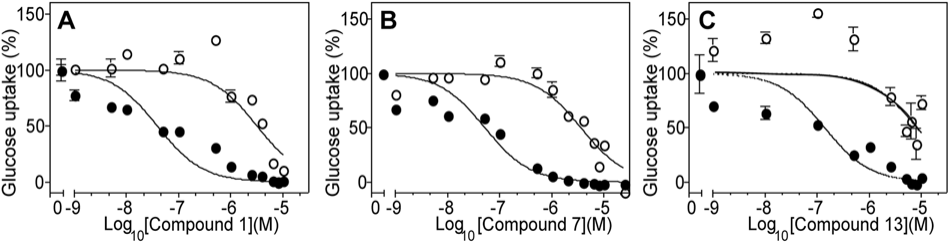
Dose-response curves for inhibition of glucose uptake by PfHT and GLUT1. Compounds 1 (A), 7 (B), and 13 (C) were applied over a range from 10^-9^–10^-5^ M to Δ*lmxgt1-3* null mutants expressing either PfHT (filled circles) or GLUT1 (open circles), and uptake of 100 μM [^3^H] D-glucose was measured in a 1 min uptake assay. Results are plotted as the mean and standard deviation (error bars) from 3 replicate uptake determinations. Data were fitted to a sigmoidal inhibition curve.

The compounds in Fig 2 are selective inhibitors of glucose versus proline uptake when glucose uptake is mediated by PfHT. To determine whether any of these compounds are selective inhibitors of PfHT versus the human glucose transporter GLUT1, we performed dose-response curves for inhibition of uptake of 100 μM [^3^H] D-glucose using the PfHT and GLUT1 reporter lines (Figs 2 and 3). Fig 2 reveals that there was one compound from the TCAMS library, TCMDC-125163 or Compound 1, and one from the Malaria Box library, GNF-Pf-3184 or Compound 7, that potently inhibited glucose uptake by PfHT (IC_50_ < 50 nM) but weakly inhibited uptake by GLUT1 (IC_50_ > 3 μM), providing respectively an 82-fold and 71-fold selective inhibition of PfHT versus GLUT1. Hence, these two compounds are high potency selective inhibitors of PfHT versus GLUT1.

**Fig 4:**
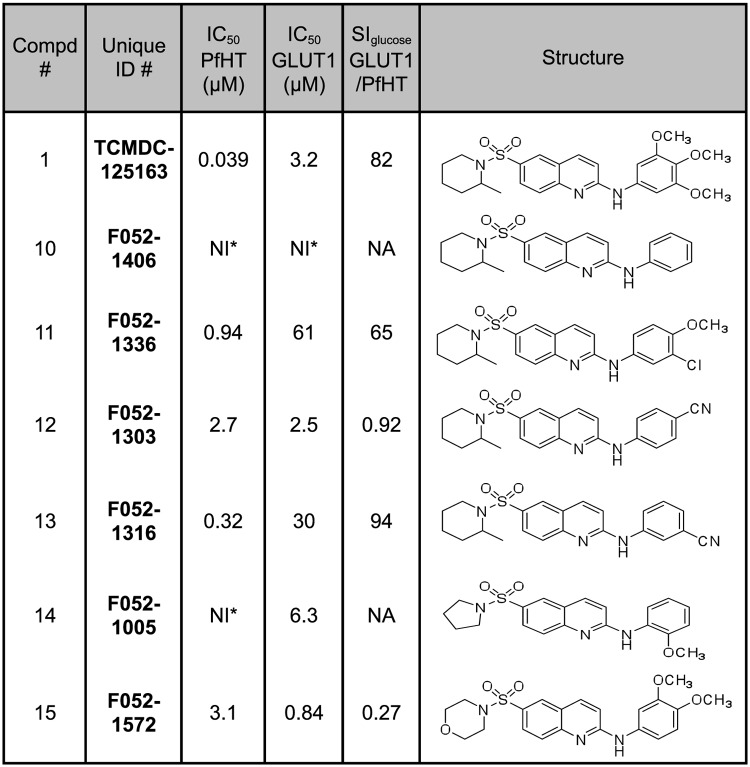
Analogs of Compound 1 were tested for inhibition of uptake of 100 μM [^3^H] D-glucose by PfHT and GLUT1, as in Fig 2. NI indicates no inhibition, and NA indicates does not apply. Other abbreviations are as in Fig 2.

**Fig 5:**
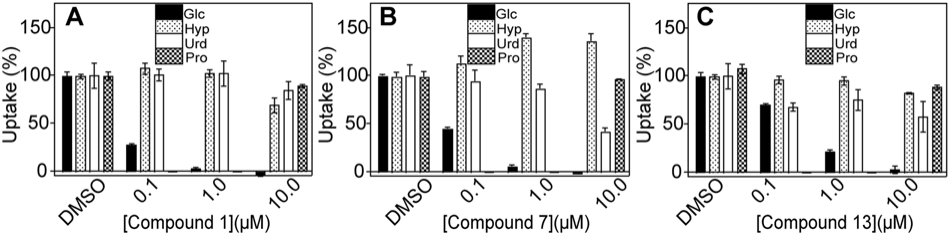
Inhibition of uptake of 100 μM [^3^H] D-glucose (Glc, black bars), 12.5 μM [^3^H] hypoxanthine (Hyp, stippled bars), 1 μM [^3^H] uridine (Urd, white bars), and 100 μM [^3^H] L-proline (Pro, square-hatched bars) by Compounds 1 (A), 7 (B), and 13 (C). Concentrations of each inhibitor are shown on the x-axis; DMSO represents the control using the DMSO vehicle without any inhibitor. Proline uptake was not measured at 0.1 or 1.0 μM concentrations of inhibitors, hence the cross-hatched bar representing proline uptake does not appear for these concentrations.

To begin to establish structure-activity relationships (SAR) for the top selective inhibitors, we performed dose-response curves for 6 analogs of Compound 1 (Fig 4). Two Compound 1 analogs, Compounds 11 and 13, did exhibit selectivity toward PfHT (65-fold and 94-fold, respectively), although their IC_50_ values for the parasite permease (0.94 and 0.32 μM, respectively) were higher than for Compound 1 (0.039 μM). Thus Compound 1 and its analogs present an encouraging SAR profile representing a range of activities over the spectrum of analogs examined, consistent with the notion that it interacts with a specific target. Both compounds strongly inhibit glucose uptake through PfHT but are poor inhibitors for uptake of hypoxanthine, uridine, or proline through the innate nucleobase, nucleoside, and proline transport systems of *L*. *mexicana* (Fig 5).

**Fig 6:**
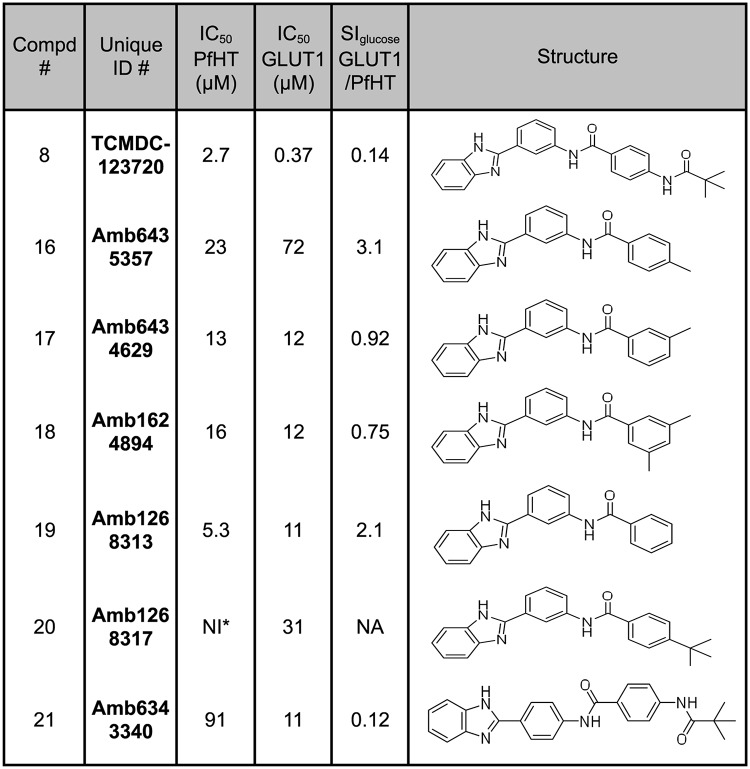
Analogs of the low potency, low specificity inhibitor Compound 8 were tested for inhibition of uptake of 100 μM [^3^H] D-glucose by PfHT and GLUT1, as in Fig 2. Abbreviations are as in Figs 2 and 4.

Compounds 2–6, 8, and 9 in Fig 2 inhibit uptake of glucose through PfHT with IC_50_ values ranging from 1.1 to 27 μM; additionally, the ratio of IC_50_ values for GLUT1/PfHT ranges from 0.055 to 15. Hence, these compounds are low potency, low specificity inhibitors of PfHT. Four of them (compounds 4–6 and 8) inhibit GLUT1 better than PfHT. Some of these scaffolds might nonetheless hold promise if certain analogs of the original hits should prove to be high potency selective inhibitors of PfHT. Indeed, Fig 4 demonstrates that analogs of Compound 1 exhibit a range of potencies and specificities for PfHT versus GLUT1, suggesting that a similar pattern might apply to some of the other compounds in Fig 2. To probe this question, we examined 6 analogs of Compound 8 (Fig 6), one of the hits that has higher potency toward GLUT1 than PfHT. While none of these analogs proved to be a high potency selective inhibitor of PfHT, some of them (Compounds 16 and 19) did reverse specificity, showing somewhat better selectivity for PfHT versus GLUT1 (e.g., IC_50_ ratios for GLUT1/PfHT of 2–3 versus 0.14, a 15-22-fold improvement in specificity). To determine whether any of the scaffolds represented by Compounds 2–6, 8, and 9 could produce high affinity selective inhibitors of PfHT, it will be necessary to carry out an extensive structure-activity studies for each scaffold, studies that are beyond the scope of the current investigation.

### Kinetics for Inhibition of PfHT by Compounds 1, 7 and 13

**Fig 7:**
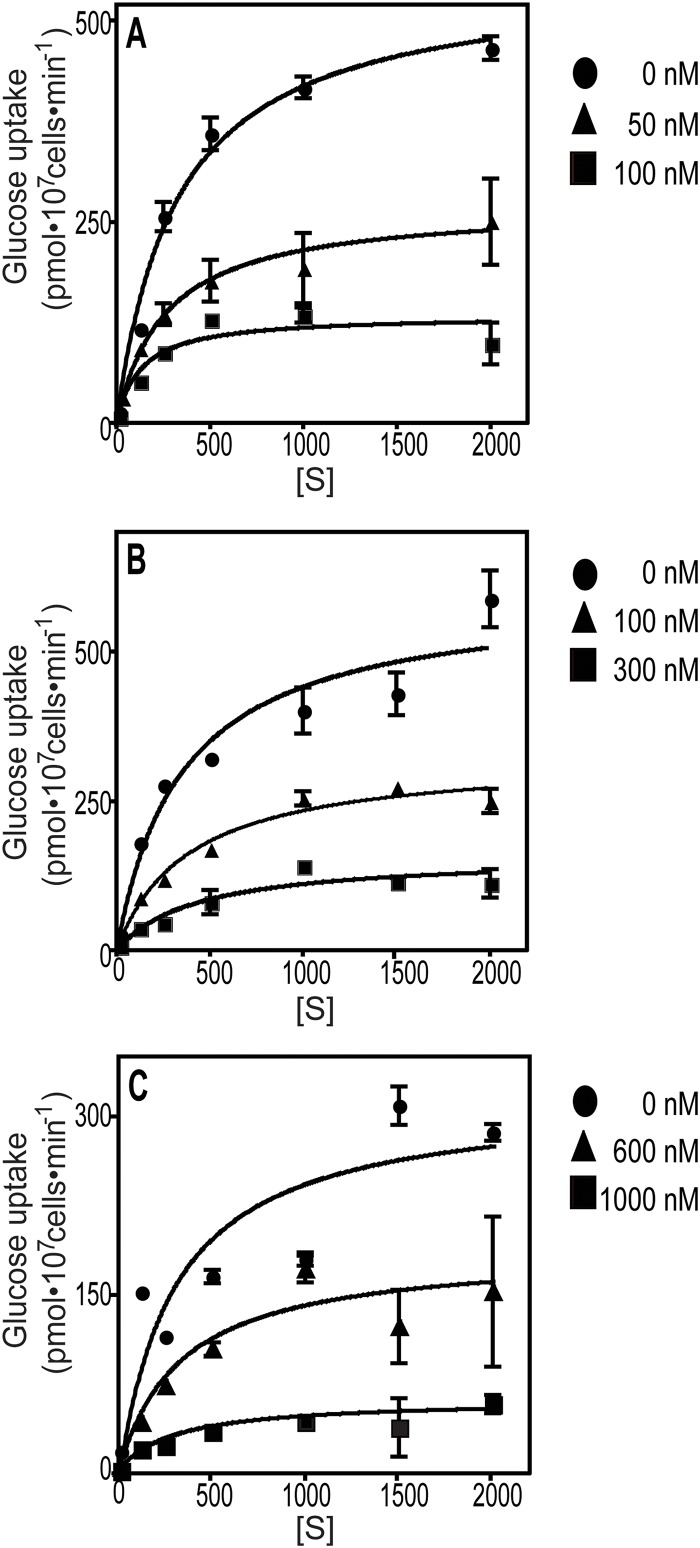
Inhibition of glucose uptake by PfHT by Compounds 1, 7, and 13. Substrate saturation curves (A, B, C) were performed for PfHT in the presence of various concentrations of Compounds 1, 7, and 13, respectively. Data represent the mean and standard deviation of 3 replicate uptake assays. Data were fitted to the Michaelis-Menten equation by non-linear regression.

To further investigate the mode of action of the top hits from the screens, we performed kinetic analysis for inhibition of glucose uptake through PfHT. Fig 7 shows substrate saturation curves for several concentrations of Compounds 1, 7, and 13. The K_m_ and V_max_ values resulting from curve fits to the Michaelis-Menten equation are tabulated in Table 1. Statistical analysis of the V_max_ and K_m_ values, determined at different concentrations of each compound, using one-way ANOVA, reveal that the V_max_ values decrease significantly as each compound concentration increases, but the K_m_ values do not differ significantly from each other as compound concentration increases. These results suggest that Compounds 1, 7, and 13 act as non-competitive inhibitors of glucose and hence likely interact at distinct sites on PfHT from the glucose binding pocket. However, most cases of nominal non-competitive inhibition are more likely to represent ‘mixed inhibition’ in which K_m_ values do change somewhat with increasing inhibitor concentration [37]. None of these compounds acts as a competitive inhibitor that would need to compete with the abundant glucose supply present in the bloodstream of a malaria-infected host. The K_ic_ and K_iu_ values (inhibition constants for binding of inhibitor to free and glucose-bound PfHT, respectively, determined graphically [37] using the K_m_ and V_max_ values in Table 1) are also in the nM range for Compounds 1, 7 and 13, indicating high affinity inhibition.

**Table 1:** The kinetics of inhibition of glucose uptake by Compounds 1, 7, and 13.

Compound	[I] (nM)	Vmax	Km (μM)	Kic (μM)	Kiu (μM)
**Compound 1**	0	573 ± 27	411 ± 123		
	50	280 ± 15	289 ± 44	0.033	0.023
	100	131 ± 4	310 ± 255	(r^2^ = 0.94)	(r^2^ = 0.97)
		p = 0.0003*	p = 0.75		
**Compound 7**	0	534 ± 87	411 ± 84		
	100	274 ± 71	403 ± 21	0.093	0.091
	300	128 ± 42	399 ± 42	(r^2^ = 0.99)	(r^2^ = 0.99)
		p = 0.022*	p = 0.97		
**Compound 13**	0	388 ± 59	375 ± 100	0.33	0.26
	600	245 ± 67	466 ± 86	(r^2^ = 0.96)	(r^2^ = 0.88)
	1000	93 ± 53	326 ± 18		
		p = 0.036*	p = 0.32		

* indicating p<0.05

The V_max_ and K_m_ values (averages and standard deviations for at least 2 determinations), determined from the substrate saturation curves such as those in Fig 7, are tabulated for compounds 1, 7, and 13. Values of K_m_ (μM) and V_max_ (pmol per 10^7^ cells per min) are given for each concentration of inhibitor [I]. Values were obtained by fitting the data to the Michaelis-Menten equation by non-linear regression. One-way ANOVA was employed to infer whether the K_m_ and V_max_ values, determined at different concentrations of each compound, were significantly different from each other (* indicating p<0.05). K_i_ values were determined by plotting K_m_/V_max_ versus [I] to obtain the K_ic_ (K_i_ for binding of inhibitor to free transporter) and 1/V_max_ versus [I] to obtain the K_iu_ (K_i_ for binding of inhibitor to substrate-bound transporter), as described in reference [37]. Data were fitted by linear regression (r^2^ values associated with each K_i_ refer to fits of the line to the data), and K_i_ values were obtained from the negative of the x-intercept.

### Inhibition of Growth of Intraerythrocytic *P*. *falciparum in vitro*

To determine whether selective inhibitors of PfHT are effective against malaria parasites *in vitro*, Compounds 1, 10, 12, and 13 were tested in dose-response format for their ability to inhibit proliferation of intraerythrocytic *P*. *falciparum* parasites (drug sensitive 3D7 and multi-drug resistant K1 strains, Table 2). The most potent inhibitor of PfHT, Compound 1, was also the most potent inhibitor of proliferation, with an EC_50_ of 1.4 μM and 0.97 μM respectively for 3D7 and K1 strains. In contrast, Compounds 10, 12, and 13 that are much poorer inhibitors of PfHT exhibited EC_50_ values of >10 μM for growth inhibition. None of the 4 analogs showed substantial toxicity toward human foreskin fibroblasts (BJ cells). Although the number of compounds examined was limited, compound 1 that exhibited the highest potency and selectivity for inhibition of PfHT also possessed the highest potency for inhibition of *P*. *falciparum* growth in vitro.

**Table 2:** Inhibition of proliferation of malaria parasites and of human foreskin fibroblasts by Compounds 1, 10, 12, and 13 and control compounds.

Compound	Pf 3D7 EC_50_ (μM)	95% Cl	Pf K1 EC_50_ (μM)	95% Cl	BJ EC_50_ (μM)	95% Cl
**Compound 1**	1.4	0.72–2.7	0.97	0.58–1.6	>23	ND
**Compound 10**	15	13–17	12	5.0–29	>25	ND
**Compound 12**	>16	ND	>16	ND	>26	ND
**Compound 13**	11	5.6–20	7.3	6.0–8.9	>25	ND
**Mefloquine**	0.12	0.11–0.13	0.020	0.020–0.030	ND	ND
**Chloroquine**	0.080	0.010–0.45	0.81	0.66–0.99	ND	ND
**Gambogic acid**	ND	ND	ND	ND	2.5	1.6–4.0

Dose response curves were performed for Compound 1 and three of its analogs to quantify inhibition of proliferation of *P*. *falciparum* strains 3D7 (not drug resistant) and K1 (multi-drug resistant) and human foreskin fibroblasts (BJ cells). The numbers under each cell line refer to the EC_50_ values in μM units. Columns marked 95% Cl refer to the 95% confidence limits for determination of EC_50_ values. Control experiments were also performed with the anti-malarial compounds mefloquine and chloroquine and with gambogic acid, which inhibits proliferation of mammalian cells. ND indicates not done.

### *In vitro* Pharmacokinetic Profiles

The solubility and permeability of the top hit Compound 1 and its analogs (Table 3), were measured using *in vitro* assays in order to determine likelihood of oral absorption. Likewise, metabolic half-life following exposure to mouse liver microsomes was studied to predict liver clearance *in vivo*. Compound 1 exhibits low aqueous solubility, high membrane permeability, but also relatively high membrane retention, and relatively high rate of metabolism by liver microsomes. Thus, while exhibiting strong performance in cellular assays, the compound series will require further optimization prior to validation *in vivo*.

**Table 3:** *In vitro* absorption-distribution-metabolism-excretion (ADME) properties of Compounds 1, 10, 12, 13 and control compounds.

Compound	F.W.	Solubility (μM)	Permeation (10^-6^ cm/s)	Retention (%)	Mouse Metabolic Stability t1/2 (hr)	Clint (mL/min/Kg)
**Compound 1**	471.6	0.50 ± 0.40	1057 ± 69	70 ± 2.0	0.27 ± 0	213
**Compound 10**	381.5	0.40 ± 0	534 ± 25	80 ± 12	0.13 ± 0	438
**Compound 12**	406.5	0 ± 0	570 ± ND	93 ± 3.8	0.32 ± 0	178
**Compound 13**	406.5	0.90 ± 1.2	582 ± 205	93 ± 0.5	0.080 ± 0	680
**Albendazole**	265.3	5.9 ± 2.3	286 ± ND	59 ± 27	ND	ND
**Carbamazepine**	236.3	87 ± 1.2	85 ± 21	43 ± 3.0	ND	ND
**Ranitidine HCl**	350.9	84 ± 1.2	0 ± 0	1.7 ± 1.3	ND	ND
**Verapamil HCl**	491.1	77 ± 3.2	1053 ± ND	74 ± 7.8	0.86 ± 0.10	67

Aqueous solubility, permeation and retention in artificial membrane, metabolic stability against mouse liver microsomes (t_1/2_ or half-life), and intrinsic clearance (Clint) were determined as described in Materials and Methods. Values were also determined for albendazole, carbamazepine, ranitidine, and verapamil as control drugs. ND indicates not done.

## Discussion

In this study, focused libraries of compounds with demonstrated proliferation inhibitory capacity against *P*. *falciparum* intraerythrocytic stage parasites were employed, because any hits would constitute compounds with demonstrated efficacy against the parasite. Screening of the TCAMS and Malaria Box libraries each generated one PfHT selective inhibitor, and characterization of several analogs uncovered another selective inhibitor with 8-fold lower affinity, Compound 13 in which the three methoxy groups of Compound 1 have been replaced by a *meta*-cyano group. These results warrant further examination of additional Compound 1 analogs to search for additional derivatives of this scaffold that might have higher affinity, increased selectivity, or improved physico-chemical properties as a lead for further drug development.

In addition, the initial SAR studies on Compound 1 revealed a range of potencies and specificities for different analogs (Fig 4). These results suggest the possibility that some analogs of other low potency, low specificity inhibitors (Fig 2) might emerge as structurally related high potency, high specificity compounds. The advantage of this approach is that it could expand the number of scaffolds representing selective inhibitors of PfHT that might be advanced as alternate drug leads. A variety of commercially available analogs exist for each of the compounds in Fig 2, and these can be tested for inhibition of PfHT and antimalarial activity. Indeed, a limited SAR study of 6 analogs of Compound 8 (Fig 6) did detect compounds with improved specificity for PfHT, suggesting that further SAR on this and other scaffolds may be warranted.

Currently, the most promising scaffold for further development is represented by Compound 1, for which the structure should allow facile modification to pursue a medicinal chemistry program. Similar advantages apply to other scaffolds represented in Fig 2, should high affinity selective inhibitors emerge from initial SAR studies. In contrast, Compound 7 probably represents a probe-like rather than a drug-like compound, that is, a compound with chemical and structural properties that are not optimal for drug development but may be useful for biochemical analysis. Nonetheless, the identification of multiple selective inhibitors of PfHT provides proof of principle that non-substrate analogs can provide potent antagonists that act at sites different from those occupied by substrates.

Compound 1 inhibits proliferation of both multi-drug resistant and non-resistant intraerythrocytic *P*. *falciparum* with EC_50_ values of ~1 μM (Table 2). In contrast, Compounds 10, 12, and 13, which are all less potent inhibitors of glucose uptake through PfHT, inhibit growth of parasites *in vitro* with significantly weaker potency. The correlation between inhibition of PfHT and growth suppression suggests that Compound 1 may exert its proliferation inhibitory effect through inhibition of PfHT, as anticipated for a compound that impairs the ability of the parasite to acquire an essential nutrient. Hence, PfHT is a potential pharmacological target of Compound 1, although rigorous demonstration of this point will require further studies.

The *in vitro* pharmacokinetic and physical properties of Compound 1 (Table 3) reveal that is has relatively low aqueous solubility and high membrane permeability but also a high percentage of retention in the membrane, when compared to the range of properties for the four control drugs. The metabolic stability is relatively low and the intrinsic clearance is relatively high, compared to verapamil. The high membrane permeability and low solubility place these compounds in Biopharmaceutics Class II [38] and indicates that such compounds would likely require appropriate formulation to provide reasonable oral bioavailability. These properties also suggest that for Compound 1 to be progressed toward a potential anti-malarial lead, a medicinal chemistry program would need to focus on decreasing membrane retention and increasing metabolic stability. Alternate scaffolds may emerge if any of the analogs of low potency, low specificity PfHT inhibitors exhibit higher potency and specificity toward PfHT. This approach could provide multiple scaffolds of diverse structure that could be explored for optimal drug-like properties.

Overall this study confirms that it is possible to identify high affinity, high selectivity inhibitors of PfHT by screening focused libraries of drug-like compounds that inhibit growth of *P*. *falciparum*. Similarly, screens of larger unfocused libraries may identify other such PfHT inhibitors that could be optimized subsequently for anti-malarial activity. This work further suggests that PfHT may have potential as a novel drug target for control of this pathogen of global importance.
